# Placental location, postpartum hemorrhage and retained placenta in women with a previous cesarean section delivery: a prospective cohort study

**DOI:** 10.1080/03009734.2017.1356405

**Published:** 2017-08-22

**Authors:** Johanna Belachew, Karin Eurenius, Ajlana Mulic-Lutvica, Ove Axelsson

**Affiliations:** a Department of Women’s and Children’s Health, Uppsala University, Uppsala, Sweden;; b Centre for Clinical Research Sörmland, Uppsala University, Uppsala, Sweden

**Keywords:** Postpartum hemorrhage, previous cesarean section, retained placenta, ultrasound, vascularization index

## Abstract

**Objective:**

Women previously giving birth with cesarean section have an increased risk of postpartum hemorrhage (PPH) and retained placenta. The objective of this study was to determine if anterior placental location increased the risk of PPH and retained placenta in such women.

**Materials and methods:**

We performed a prospective cohort study on 400 women with cesarean section delivery in a previous pregnancy. Ultrasound examinations were performed at gestational week 28–30, and placental location, myometrial thickness, and three-dimensional vascularization index (VI) were recorded. Data on maternal age, parity, BMI, smoking, gestational week at delivery, induction, delivery mode, oxytocin, preeclampsia, PPH, retained placenta, and birth weight were obtained for all women. Outcome measures were PPH (≥1,000 mL) and retained placenta.

**Results:**

The overall incidence of PPH was 11.0% and of retained placenta 3.5%. Twenty-three women (11.8%) with anterior placenta had PPH compared to 12 (6.9%) with posterior or fundal locations. The odds ratio was 1.94, but it did not reach statistical significance. There was no significant risk increase for retained placenta in women with anterior placentae. Seven of eight women with placenta previa had PPH, and four had retained placenta.

**Conclusions:**

The overall risk of PPH and retained placenta was high for women with previous cesarean section. Anterior location of the placenta in such women tended to impose an increased risk for PPH but no risk increase of retained placenta. Placenta previa in women with previous cesarean section is associated with a high risk for PPH and retained placenta.

## Introduction

As rates of cesarean section continue to increase worldwide (1), methods for prediction, surveillance, and management of complications during pregnancy and delivery associated with previous cesarean section become increasingly important. Uterine rupture, placenta previa, and placenta accreta are well-known and potentially life-threatening complications, but are fortunately still rare conditions (2,3). They have, however, increased alongside the rising number of women with previous uterine surgery (4). A decision-analytic model by Solheim et al. (5) has predicted a substantial increase in the incidence of placenta previa and accreta and in maternal mortality if the cesarean section rate continues to increase.

Postpartum hemorrhage (PPH) is more common and is associated with maternal mortality and morbidity. Women with previous cesarean section delivery have a higher risk for PPH compared with women without previous cesarean section (6,7). The incidence of PPH has also increased during the last decades (8,9).

We have previously conducted a population-based cohort study that showed an increased risk (3.44%) of retained placenta in women previously delivered by cesarean section (10). The risk was higher for retained placenta with PPH than for retained placenta with normal blood loss. The reasons for this increased risk are not fully understood, but theories focus on the scarred uterine wall and resemble discussions concerning the pathology behind placenta previa and accreta (11). If the abnormal implantation in the uterine wall is caused by the cesarean section scar, the placenta should be located over the scarred myometrium in order to be retained and cause PPH. It has been reported that, among women with placenta previa, all with abnormal invasive placentae had a previous cesarean section and anterior placenta previa (12). Pictorial ultrasound, including measurements of myometrial thickness and 3D power Doppler at the placental site, has been used to diagnose invasive placentation (12,13).

In Sweden, every pregnant woman is offered a routine second trimester ultrasound at 18 weeks of gestation, and 97% of all women attend (14). Gestational age is estimated, fetal anomalies looked for, and location of the placenta is recorded. In case of a low-lying placenta the woman is examined again in the third trimester to evaluate if the placenta has migrated or not. Previous studies have shown that migration of a low-lying placenta occurred less often in women with previous cesarean section (15). Whether or not there is an association between the location of the placenta in a scarred uterus and complications such as retained placenta and PPH has not yet been examined. Therefore, the aim of this study was to investigate if an anterior placental location in women with a previous cesarean section delivery increased the risk for PPH and retained placenta.

## Materials and methods

This prospective longitudinal study was performed between July 2010 and October 2013, at the Department of Obstetrics and Gynecology, Uppsala University Hospital, Sweden. Uppsala University hospital is a tertiary referral center with approximately 4,000 deliveries per year. The cesarean section rate during the study period was 16%.

All pregnant women who had been delivered by cesarean section in at least one previous pregnancy were eligible for participation. They were invited to participate by the midwife conducting the routine ultrasound at approximately gestational week 18. Only singleton pregnancies were included. Reasons for non-participation were not recorded. The women were examined by abdominal ultrasound at gestational week 28–30 by one of three physicians (J.B., K.E., A.M.L.) using Voluson E8 (GE Medical Systems) ultrasound equipment. This time period was chosen in order not to exclude preterm deliveries. The location of the placenta was recorded as anterior, low anterior, posterior, low posterior, fundal, posterior previa, or anterior previa. Low anterior or posterior was defined as placenta localized close to but more than 2 cm from the internal cervical os. Previa was diagnosed when the placenta was less than 2 cm from or covered the cervical os. The thickness of the myometrium at the midsection of the placenta was determined. Vascularization index (VI) measured by 3D ultrasound power Doppler was obtained from a preset sonobiopsy in the midsection of the placenta and uterine wall. Data on age, body mass index (BMI), smoking, parity, and number of previous cesarean sections were recorded for all women. Data on induction, mode of delivery, use of oxytocin to promote labor, gestational week at delivery, infant birth weight, retained placenta, and PPH were retrieved from the records. Blood loss at delivery was estimated by the delivering midwife or obstetrician and documented in the women’s patient record. If the bleeding was estimated to exceed 500 mL all cloths and sheets were weighed for more exact measurement. Main outcomes were PPH defined as blood loss ≥1,000 mL and retained placenta. Retained placenta was diagnosed when placenta had to be removed manually after vaginal delivery or was substantially difficult to remove during cesarean section. The clinicians who diagnosed PPH and retained placenta had no knowledge on the outcome of the ultrasound scan at week 28.

### Statistical analysis

All statistical analyses were performed using SPSS statistical software, version 19. For PPH and retained placenta as the outcome variables, crude and adjusted odd ratios (OR) were used to calculate the association with placental location. The adjusted ORs were estimated by logistic regression. When calculating the association between VI and amount (mL) of blood loss, Pearson’s correlation coefficient was used. A *P* value of less than 0.05 was considered statistically significant.

### Power calculation

The incidence of PPH was presumed to be 10%. To detect an increase to 20% in women with anterior placentae with a two-sided alfa of 0.05 and a beta of 0.20, 398 women had to be included. Therefore, recruitment was stopped when 400 women were included.

The regional ethical board at the Medical Faculty, Uppsala University, Sweden approved the study, and informed consent was obtained from all participants.

## Results

During the study period about 900 women with previous cesarean section had their routine scan, but limited personnel resources led to periods of low recruitment. Of the 529 women who were invited to participate, 407 agreed. Seven women were excluded; five because of delivery before week 28 and two because they had moved before week 28.

There were minor or no differences in maternal, delivery, and infant characteristics between participants and non-participants (Table 1). Among the non-participants more had more than one previous cesarean section, more were multiparous, more were smokers, and fewer had induced labor. At 28 weeks 218 (54.5%) of the placentae were anterior, and 182 (45.5%) had other locations (Table 2).

**Table 1.** Maternal, delivery, and infant characteristics in participants and non-participants.

	Participants (*n* = 400)	Non-participants (*n* = 129)
Age (years)	
22–29	75 (18.8%)	28 (21.7%)
30–39	288 (72.0%)	90 (69.8%)
40–49	37 (9.2%)	11 (8.5%)
Parity (para)	
1	272 (68.3%)	75 (58.1%)
>1	126 (31.7%)	54 (41.9%)
Previous cesarean section	
1	337 (85.1%)	102 (79.0%)
>1	59 (14.9%)	27 (21.0%)
Body mass index	
18–25	233 (58.8%)	77 (63.1%)
26–30	109 (27.6%)	27 (22.1%)
31–53	54 (13.6%)	18 (14.8%)
Smoking, Yes	15 (3.8%)	8 (6.3%)
Preeclampsia, Yes	10 (2.5%)	1 (0.8%)
Gestational week at delivery	
31–36	22 (5.5%)	10 (8.1%)
37–42	376 (94.5%)	113 (91.9%)
Mode of delivery	
Vaginal, non-instrumental	145 (36.3%)	50 (39.0%)
Vaginal, vacuum extraction	34 (8.5%)	8 (6.3%)
Caesarean section	221 (55.2%)	70 (54.7%)
Induction, Yes	66 (16.7%)	11 (8.7%)
Use of oxytocin, Yes	125 (31.4%)	34 (26.6%)
Birth weight (g)	
Mean	3723	3589
Range	1687–5090	2010–4840
Myometrial thickness (mm)	
Mean	5.5	
Range	3.0–10.5	
Vascularization index (%)	
Mean	25.8	
Range	2.5–63.0	
Postpartum hemorrhage (mL)	
<1000	356 (89.0%)	107 (85.6%)
≥1000	44 (11.0%)	18 (14.4%)
Retained placenta, Yes	14 (3.5%)	6 (4.7%)

**Table 2.** Placental location at 28 weeks.

Placental location	*n*	Percent
Anterior	195	48.7%
Low anterior	17	4.3%
Posterior	152	38.0%
Low posterior	7	1.7%
Fundal	21	5.3%
Anterior previa	6	1.5%
Posterior previa	2	0.5%
Total	400	100%

Between the routine scan at 18 weeks and the examination at 28 weeks, 23 (62.2%) of low-lying placentae had migrated to a posterior or anterior position. Eight women (2.0%) were diagnosed with placenta previa; two were posterior and six anterior. Three women with anterior placenta previa were diagnosed as placenta accreta by ultrasound, and two of them had a hysterectomy performed at delivery. The third woman did not have a hysterectomy, and blood loss was only 800 mL. Four women (1.0%) were diagnosed with uterine rupture.

The overall incidence of PPH was 11.0% and of retained placenta 3.5% (Table 1).

Twenty-three women (11.8%) with anterior placenta had PPH compared to 12 (6.9%) with posterior or fundal placenta (Table 3). The adjusted OR was 1.94, but the difference was not statistically significant (*P* = 0.10); the corresponding figure when excluding cases with uterine rupture was 2.13 (*P* = 0.06). Among the women with retained placenta three (1.5%) were anterior and seven (4.0%) localized elsewhere. Adjustments were made for maternal age, parity, number of previous cesarean sections, gestational age at delivery, mode of delivery, induction, use of oxytocin, and infant birth weight. Among the women with low-lying anterior or posterior placenta there was only one woman with PPH in each group and none with retained placenta. Five of the six women with anterior placenta previa and both of the two with posterior placenta previa had PPH. Three of the six women with anterior previa and one of the two with posterior previa had retained placenta (Table 3).

**Table 3.** Odds ratios for PPH (PPH > 1,000 mL) and retained placenta correlated to placental location.

	PPH				Retained placenta			
			Odds ratio				Odds ratio	
Placental location	*n* (total)	Rates, %	Crude	Adjusted	*n* (total)	Rates, %	Crude	Adjusted
Anterior	23 (195)	11.8	1.79 (*P* = 0.12)	1.94 (*P* = 0.10)	3 (195)	1.5	0.37 (*P* = 0.16)	0.34 (*P* = 0.13)
Posterior or fundal	12 (173)	6.9	reference	reference	7 (173)	4.0	reference	reference
Low anterior	1 (17)	5.9	0.34 (*P* = 0.51)	–	0 (17)	0	–	–
Low posterior	1 (7)	14.3	reference	reference	0 (7)	0	reference	reference
Anterior previa	5 (6)	83.3	–	–	3 (6)	50	1 (*P* = 1.0)	–
Posterior previa	2 (2)	100	reference	reference	1 (2)	50	1	–

Adjusted for: maternal age, parity, number of previous caesarean sections, gestational age at delivery, mode of delivery, induction, use of oxytocin, and birth weight.

Vascularization index (VI) ranged from 2.5% to 63.0% with a mean of 25.8%. There was no difference between anterior and posterior placenta. However, the examiners found the 3D volumes with VI easier to access in anterior placenta. There was no increase in VI in cases of PPH or retained placenta. Myometrial thickness did not correlate with PPH or retained placenta (data not shown).

## Discussion

The frequency of PPH for women with anterior placenta tended to be higher, although not significantly, than that for other placental locations (11.8% compared to 6.9%). An increased risk of PPH and retained placenta for women with previous cesarean section and placenta previa was verified in anterior as well as posterior placenta previa. VI or myometrial thickness at the placental site was not associated with PPH or retained placenta.

The main strength of the study is its prospective longitudinal design. All examinations were performed by the authors (J.B., K.E., A.M.L.) in the department where the women were delivered, optimizing reliability of the data. The substantial proportion of women previously delivered by cesarean section who did not participate in the study is a limitation. However, we found only minor differences concerning background data and outcomes between participants and non-participants. The reasons for lack of recruitment were purely logistic in nature and should not have caused selection bias.

We found a PPH incidence of 11.9% in this group of women with previous caesarean section delivery. This is in agreement with Taylor et al. (7) who report a figure of 8.7%. In a general population, incidences of 2.8% (6) and 2.6% (8) have been reported. Retained placenta occurred in 3.8%, which is well in line with our previous population-based study (10).

There was higher rate of PPH in women with anterior placenta, but the difference did not attain statistical significance. The present study was powered to detect a risk increase of 10%.

The incidence of placenta previa varies between different reports. Our finding, 2.0%, for women with at least one prior cesarean section is similar to that previously reported by To et al. (1.31%) (16) but substantially lower than the 5% reported by Jauniaux and Jurkovic (17). We could not confirm the results of Naji et al. (18) stating that more placentae are posterior in women with a previous cesarean section. On the contrary, we found a majority of the placentae to be anterior, which also holds true for placenta previa. The reason for this difference might be that Naji et al. (18) assessed placental location at week 12 and we at week 28. Our figure, 5.3%, for fundal placentae is, however, well in line with the 4.7% reported by Naji et al. (18). Moreover, the percentage of placental migration from a low-lying position is in the same range, about 60%, as that found by Naji et al. (18), although the interval between the observations differs. Interestingly, Lal et al. (15) have reported that 61% of second trimester placenta previa in women with a prior cesarean section have migrated before delivery.

Assuming that retained placenta and PPH in women previously delivered by cesarean section share the same mechanism as placenta accreta, although less invasive, the antenatal diagnostic signs might be similar. Many reports have evaluated different diagnostic signs for placenta accreta (19). Wong et al. (13) found myometrial thickness less than 1 mm predictive, and Cali et al. (12) found irregular hypervascularization, seen by 3D ultrasound, in cases of placenta accreta. We found no association between myometrial thickness and PPH or retained placenta. Moreover, we found no difference in VI between women with PPH and others. Our findings cannot, however, be directly compared to those by Cali et al. since they examined only cases of placenta previa, which made it possible to measure the uterine serosa–bladder wall interface. In our study, we measured the VI on all placental locations. In week 28, it is not possible to measure the entire placental width. Therefore, we used a sonobiopsy to evaluate placental vascular indices, which has been shown to correlate well to 3D power Doppler of the entire placenta (20). However, other studies have shown poor reliability and reproducibility when estimating placental perfusion using 3D power Doppler (21,22).

Our findings showing an increased risk for PPH and retained placenta in women with placenta previa are not surprising, but we stress the importance of a correct prenatal diagnosis of placental location. This is a prerequisite to optimize the management at delivery. Cases not diagnosed prenatally have an increased risk of maternal morbidity (23). Moreover, the high rate of PPH, retained placenta, placenta previa, and uterine rupture, shown here in women with a previous caesarean section, adds further important knowledge to the highly topical discussion on the increasing rates of caesarean section worldwide.
